# Charging Scheduling Method for Wireless Rechargeable Sensor Networks Based on Energy Consumption Rate Prediction for Nodes

**DOI:** 10.3390/s24185931

**Published:** 2024-09-12

**Authors:** Songjiang Huang, Chao Sha, Xinyi Zhu, Jingwen Wang, Ruchuan Wang

**Affiliations:** School of Computer Science, Software and Cyberspace Security, Nanjing University of Posts and Telecommunications, Nanjing 210003, China; jim_songjiang@163.com (S.H.); b21030106@njupt.edu.cn (X.Z.); wjw1072045262@163.com (J.W.); wangrc@njupt.edu.cn (R.W.)

**Keywords:** wireless rechargeable sensor networks, charging scheduling method, event missing rate minimization, energy consumption rate prediction, network topology construction

## Abstract

With the development of the IoT, Wireless Rechargeable Sensor Networks (WRSNs) derive more and more application scenarios with diverse performance requirements. In scenarios where the energy consumption rate of sensor nodes changes dynamically, most existing charging scheduling methods are not applicable. The incorrect estimation of node energy requirement may lead to the death of critical nodes, resulting in missing events. To address this issue, we consider both the spatial imbalance and temporal dynamics of the energy consumption of the nodes, and minimize the Event Missing Rate (EMR) as the goal. Firstly, an Energy Consumption Balanced Tree (ECBT) construction method is proposed to prolong the lifetime of each node. Then, we transform the goal into Maximizing the value of the Evaluation function of each node’s Energy Consumption Rate prediction (MEECR). Afterwards, the setting of the evaluation function is explored and the MEECR is further transformed into a variant of the knapsack problem, namely “the alternating backpack problem”, and solved by dynamic programming. After predicting the energy consumption rate of the nodes, a charging scheduling scheme that meets the Dual Constraints of Nodes’ energy requirements and MC’s capability (DCNM) is developed. Simulations demonstrate the advantages of the proposed method. Compared to the baselines, the EMR was reduced by an average of 35.2% and 26.9%.

## 1. Introduction

With the rapid development of the Internet of Things (IoT), Wireless Sensor Networks (WSNs) have found extensive applications in fields such as environmental monitoring [1], smart homes [2], and healthcare [3]. However, the limited battery capacity of sensor nodes poses a significant challenge to their long-term deployment and operation. To address this issue, Wireless Rechargeable Sensor Networks (WRSNs) have been developed. One or more Mobile Chargers (MCs) equipped with Wireless Power Transfer (WPT) technology are adopted to enable the remote and wireless charging of nodes, thereby significantly extending the network lifetime [4,5,6,7,8,9].

The energy consumption rate of nodes is often dynamic, fluctuating with the occurrence of events. Moreover, nodes closer to the Base Station (BS) bear a higher data burden and thus consume more energy. Without an effective charging scheduling strategy to promptly recharge these critical nodes, the network’s reliability can deteriorate rapidly, leading to potential paralysis. For example, in a nuclear wastewater monitoring network [10], if a critical sensing or forwarding node died due to energy depletion, the event reports may not be sent to the BS, causing the loss of critical data.

Most existing charging scheduling methods often overlook the temporal dynamics of node energy consumption rate, making them unsuitable for practical applications. Therefore, we propose a highly reliable charging scheduling method for WRSNs. We define the Event Missing Rate (EMR) as the ratio of the number of events not received by the base station to the total number of events occurring within the network monitoring area. Our method integrates network topology optimization and node energy consumption rate prediction, aiming to minimize the EMR.

There are three primary challenges in achieving this objective. Firstly, optimizing the network topology to facilitate effective charging scheduling is complex, even with the availability of mature routing protocols. Secondly, it is difficult to accurately predict the nodes’ energy consumption rate due to their spatial imbalance and temporal variability. Finally, selecting the nodes to charge, and determining the amount of energy supplied to them as well as the charging sequence are all complex problems for the energy-limited MC.

To address these challenges, we first propose an Energy Consumption Balanced Tree (ECBT) construction method. Then, we decompose the issue into two subproblems: (1) Maximizing the value of the Evaluation function of each node’s Energy Consumption Rate prediction (MEECR) by dynamic programming, and (2) developing a charging scheduling scheme that meets the Dual Constraints of Nodes’ energy requirements and MC’s capability (DCNM), thereby minimizing the EMR. The main contributions of this paper are summarized as follows.

- To the best of our knowledge, we are the first to jointly consider the distributional imbalance and temporal dynamics of the node energy consumption rate in WRSNs and propose a charging scheduling method that minimizes the Event Missing Rate.
- To prolong the lifetime of each node, we propose an energy consumption balanced tree construction method based on a greedy algorithm to form the network topology.
- To solve the MEECR problem, we establish an energy consumption rate prediction model and transform it into an “alternating backpack” problem, which is then solved using a dynamic programming method. To solve the DCNM problem, we propose a charging scheduling scheme that satisfies the dual constraints.

The rest of this paper is organized as follows. In Section 2, we review related works focusing on the requirements of nodes, the performance of the MC, and the quality of service. The network model and energy consumption rate prediction are introduced in Section 3. Section 4 presents the proposed construction of the energy consumption balanced tree. Section 5 provides the solution for the energy consumption rate prediction for nodes. In Section 6, the charging scheduling scheme consistent with the MC’s service capability and nodes’ energy requirements is described. The simulation results are presented and analyzed in Section 7. Section 8 concludes the paper.

## 2. Related Works

In this section, we introduce the existing works focusing on the requirements of nodes [11,12,13,14,15,16,17,18], the performance of the MC [19,20,21,22,23,24,25], and the quality of service [26,27,28,29,30,31,32] to illustrate the significance of our study.

### 2.1. Focus on the Requirements of Nodes

In WRSNs, nodes are usually regarded as the service recipients. Wang et al. [11] proposed a hybrid energy supply framework combining solar energy and wireless charging by optimizing cluster-head deployment and energy management to improve the energy efficiency and cost-effectiveness of wireless sensor networks. To maximize the minimum residual energy of nodes after data transmission, Baek et al. [12] proposed a Voronoi diagram-based algorithm for UAV hovering point determination and path planning. After that, MCs were increasingly considered for both charging and data collection tasks to minimize network energy consumption and extend network lifetime, as exemplified by an optimized bifunctional vehicle path method proposed in [13]. Additionally, Lu et al. [14] introduced a joint routing and charging algorithm utilizing MCs for data collection while accounting for dynamic factors such as topology changes to prolong the lifetime of WRSNs. Furthermore, Yang et al. [15] proposed a dynamic charging scheduling scheme based on the Actor-Critic Reinforcement Learning (ACRL) algorithm to improve average node lifetime. To enhance the robustness of the energy transfer model, Lin et al. [16] introduced the linear constraints and proposed a directional charging scheme to minimize charging delay. For scenarios involving multiple MCs, Jia et al. [17] developed an on-demand charging architecture based on dynamic collaboration among MCs, aiming to minimize energy void rates through static partitioning and online path planning. Similarly, to reduce charging delay, increase coverage, and enhance network survivability, Kaswan et al. [18] proposed a distributed mobile charging protocol based on game theory, formulating the mobile charging problem as a series of repetitive games among MCs.

While these studies contribute to extending network lifetime and reducing charging delay, they overlook the temporal and spatial attributes of nodes. Consequently, the importance of nodes (e.g., workload) may change and this may increase the risk of critical node failure under limited MC service capacity.

### 2.2. Focus on the Performance of the MC

Enhancing the energy efficiency of the MC has been a primary objective in many studies. In [19,20,21], the authors proposed some one-to-many charging schedules for the MC to charge all nodes in a region, focusing on obtaining the optimal staying points and traversal paths to reduce the charging and moving cost of the MC. Dai et al. [22] considered a scenario where MCs can only charge nodes at the edge of the network, proposing a rectangle-based partitioned mobility strategy and a sector-based rotation strategy to minimize the charging time and improve the energy efficiency of the MC. Liang et al. [23] introduced an improved Firefly Algorithm (IFA) to optimize the deployment of the MC, enhancing charging efficiency and reducing energy consumption. Furthermore, Wu et al. [24] proposed a charging-oriented sensor layout and scheduling strategy to address deficiencies in charging coverage and efficiency. To increase system robustness, Yang et al. [25] introduced a risk-averse charging schedule model, balancing utility and risk to ensure a highly effective energy supply in the presence of risks.

However, these approaches primarily focus on how to improve the performance of the MC but neglect the energy requirement of nodes. As a result, these methods may not be suitable for scenarios with dynamic node energy consumption. If node energy consumption exceeds expectations, the designed scheduling schemes may fail to ensure continuous network operation.

### 2.3. Focus on the Quality of Service

Nowadays, more and more works have focused on the quality of service during the charging scheduling process. In [26], with the goal of maximizing the amount of data collected per unit of energy consumption by the MC, Lyu et al. proposed a cell-based residency determination and a path planning method based on the fireworks algorithm, where the MC periodically charges and collects data from all nodes in the cell. Boukerche et al. [27] designed two scheduling schemes for latency-sensitive and latency-tolerant data collection, respectively, by first constructing a cluster-based topology and then using a heuristic algorithm to design scheduling schemes based on different delay requirement scenarios. Additionally, a scheduling scheme combining on-demand charging and data collection was proposed in [28], suitable for applications with low tolerance for data dissemination latency. MCs are also considered for direct environmental data monitoring in addition to collecting data from nodes. Lin et al. [29] proposed a heuristic hexagonal-based scheduling algorithm to periodically collect and monitor data from the entire area. For event-triggered WRSNs, Dai et al. [30] proposed a node dormancy/activation switching and charging scheduling strategy to maximize the quality of the network monitoring of stochastic events. While this mechanism ensures nodes are not inactivated, it reduces the robustness of network monitoring, leading to missed events during node dormancy and reduced monitoring quality. To address this, Aloqaily et al. [31] proposed a decentralized charging strategy based on local learning (LL) to charge nodes through dynamic energy consumption prediction, divided into an initialization phase and a charging scheduling phase. The node’s charging requirement is obtained through learning and prediction in the initialization phase, and the MC continuously collects node information in the charging scheduling phase, dynamically updating the prediction results to adjust the charging order. However, it only considers a single attribute of node energy, so in [32], Kan et al. designed a charging scheduling scheme by integrating each node’s coverage, network connectivity, residual energy, and path length to maximize the monitoring quality. However, this scheme only considers nodes generating data at a fixed rate.

The quality of service is crucial for ensuring the reliability of WRSNs. However, existing methods are not suitable for multi-hop transmission and event-triggered networks. They either consider the network topology while simplifying data generation methods [32] or consider random events while ignoring the network topology’s impact on event capture [30], which cannot guarantee event detection in practical applications. To address these issues, we propose a charging scheduling method based on energy consumption balanced tree construction and event-oriented node energy consumption rate prediction to minimize the EMR.

## 3. Network Model and Problem Description

### 3.1. Network Model

To increase the applicability of the proposed method, assume that *N* nodes (each of them is remarked as *s_i_* (*i* ∈ [1, *N*])) are randomly and uniformly deployed in a two-dimensional region, and all of them are aware of their locations. Compared to the deterministic deployment, the randomized uniform deployment is less costly and more flexible. Subsequently, the network runs according to the following two phases.

(1) Network Topology Construction Phase

**Figure 1:**
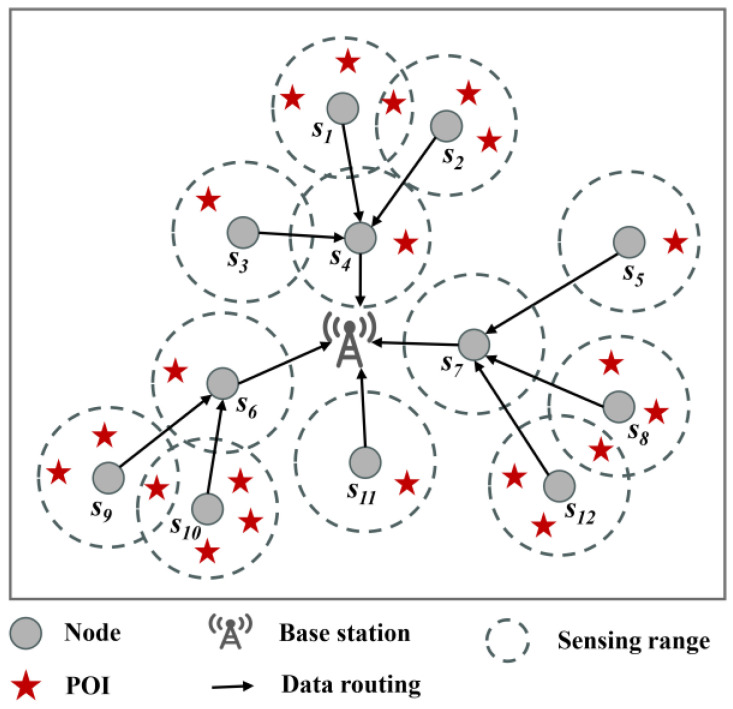
Network topology and data sensing and uploading.

As shown in Figure 1, it is a simple example of a tree network topology. Twelve nodes communicate with the BS via multi-hop transmission. To achieve a balanced load among nodes, a network topology rooted at the BS is formed based on the proposed Energy Consumption Balanced Tree (ECBT) construction method. This approach aims to reduce the node failure rate and the EMR. The construction process of the ECBT is explained in detail in Section 4.

(2) Data Uploading Phase

Once the topology is constructed, several nodes are assigned the task of monitoring the randomly deployed Points of Interest (POIs) within their sensing range. As soon as an event occurs at one of these POIs, the nodes immediately transmit the sensing data to the BS along the constructed paths, hop-by-hop, as shown by the black paths in Figure 1. It is evident that the nodes can be categorized into three types based on the tasks they perform:

- Nodes that monitor POIs and only upload their own sensing data, such as *s*_1_ in Figure 1;
- Nodes that monitor POIs and both upload their own sensing data and transmit the sensing data from other nodes, such as *s*_4_ in Figure 1;
- Nodes that do not monitor any POIs (i.e., there are no POIs within their sensing range) but transmit the sensing data from other nodes, such as *s*_7_ in Figure 1.

Without a loss of generality, we assume that the MC traverses some nodes and replenishes energy to them during a fixed duration *T*. Here, the BS is both the starting and ending point of the MC’s journey. According to the setting described above, it is evident that the energy consumption rate of the nodes will change with the occurrence and termination of events. This will directly affect the charging scheduling behavior of the MC and its energy replenishment effectiveness during each round of service time. Therefore, it needs to be characterized at a fine-grained level. To reduce complexity, a discrete method is adopted where *T* is separated into *K* equal-length “time slices”. For each time slice *k* (*k* ∈ [1, *K*]), the energy consumption rate of node *s_i_* is assumed to be a constant, denoted as *p_k_*(*s_i_*).

Apparently, it is required that each *s_i_* must survive for at least one round of service time, i.e., *T* after being charged. That is, making sure that Formula (1) holds, in which *E^arr^*(*s_i_*) represents the residual energy of *s_i_* when the MC has just arrived. *E^o^*(*s_i_*) represents the energy that *s_i_* receives from the MC this time.

(1) $$E^{arr}\left(s_{i}\right)+E^{o}\left(s_{i}\right)\geq \sum_{k=1}^{K}\left(T/K\right)\cdot p_{k}\left(s_{i}\right)$$

The set of nodes served by the MC during the *j*th traversal is defined as *O_j_*, so the total energy consumption (denoted as *E_j_*(*MC*)) and total time consumption (marked as *T_j_*(*MC*)) of the MC can be represented by Formulas (2) and (3), respectively. Here, *p^m^* and *D_j_* are the energy the MC consumes to move a unit distance and the total distance the MC moves during *T*, respectively. *p^c^* and *T^c^*(*s_i_*) represent the energy transmission power of the MC as well as the time the MC spends on charging *s_i_*, respectively. The moving speed of the MC is denoted as *v*.

(2) $$E_{j}\left(MC\right)=p^{m}\cdot D_{j}+p^{c}\cdot \sum_{s_{i}\in O_{j}}T^{c}\left(s_{i}\right)$$

(3) $$T_{j}\left(MC\right)=D_{j}/v+\sum_{s_{i}\in O_{j}}T^{c}\left(s_{i}\right)$$

Obviously, to ensure that the MC can return to the BS after each round of service, it is essential to satisfy Formulas (4) and (5). Here, *E_MC_* is the battery capacity of the MC.

(4) $$E_{j}\left(MC\right)\leq E_{MC}$$

(5) $$T_{j}\left(MC\right)\leq T$$

Furthermore, the charging efficiency of the MC to each node is uniformly set to *η*, so we have

(6) $$E^{o}\left(s_{i}\right)=\eta \cdot p^{c}\cdot T^{c}\left(s_{i}\right)$$

### 3.2. Energy Consumption Rate Prediction

Without a loss of generality, we assume that a “sensing event” may occur at any POI*_l_* and follows a Poisson process with intensity *λ_l_* [33,34]. In addition, at any given time, at most one sensing event occurs at a POI and each event is independent [30]. The range that a node *s_i_* can monitor is defined as *R*(*s_i_*). Therefore, if *s_i_* can monitor the event that occurred at a POI*_l_*, there must be the POI*_l_* ∈ *R*(*s_i_*).

Once a surviving node monitors the occurrence of an event, it continues to send sensing data to the BS until the end of the event. Obviously, replenishing energy to the corresponding nodes by the MC to ensure that they do not die is an effective means to lower the EMR. However, the network often consists of a substantial number of nodes. The energy capacity of the MC and the number of nodes it can serve each time are restricted. Consequently, improving the charging scheduling efficiency of the MC is a primary objective of WRSNs. It is easy to find that “predicting the future energy consumption rate of nodes accurately” is one of the effective means to achieve this goal. So, it is analyzed here.

The energy consumption rate of *s_i_* during a round of service time *T* (i.e., the time interval between two consecutive replenishments) can be represented by the following vector.

(7) $$\vec{p_{T}\left(s_{i}\right)}=\left\{p_{1}\left(s_{i}\right),p_{2}\left(s_{i}\right),\ldots ,p_{K}\left(s_{i}\right)\right\}$$

As previously mentioned, the nodes can be categorized into three types based on the tasks they undertake. This means that there are three different energy consumption rates for each time slice of *s_i_*. That is, there are 3*^K^* results for $\vec{p_{T}\left(s_{i}\right)}$. In order to reduce the search space, only “whether there is an event or not” is used as the basis for distinguishing the energy consumption rate of a node. If there is no sensing event, the energy consumption rate of *s_i_* is *ω*_1_, otherwise it becomes *ω*_2_(*s_i_*), which reduces the possible results of $\vec{p_{T}\left(s_{i}\right)}$ to 2*^K^*. So, $C\left(s_{i}\right)=\left\{{\vec{p_{T}\left(s_{i}\right)}}_{1},{\vec{p_{T}\left(s_{i}\right)}}_{2},\ldots ,{\vec{p_{T}\left(s_{i}\right)}}_{2^{K}}\right\}$ is used to represent the space of solutions of $\vec{p_{T}\left(s_{i}\right)}$, and $f\left({\vec{p_{T}\left(s_{i}\right)}}_{x}\right)\left|x\in \left[1,2^{K}\right]\right.$ serves as the evaluation function. The larger the value of *f*(.), the greater the probability that ${\vec{p_{T}\left(s_{i}\right)}}_{x}$ occurs in the solution space *C*(*s_i_*), i.e., the prediction of the energy consumption rate of *s_i_* in the following duration *T* is more accurate. How to reasonably characterize the evaluation function *f*(.) and how to quickly find the optimal solution ${\vec{p_{T}\left(s_{i}\right)}}_{x}$ from a 2*^K^*-sized space is the key to realize a “more accurate prediction of the future energy consumption rate of nodes”. We will explain this in detail in Section 5.

### 3.3. Problem Description

The objective of this paper is to Minimize the EMR (MEMR). Thus, based on the assumptions above, it can be split into two subproblems as follows.

- Maximizing the value of the Evaluation function (denoted as $f\left({\vec{p_{T}\left(s_{i}\right)}}_{x}\right)$) of each node’s Energy Consumption Rate prediction (MEECR problem). It is evident that precisely predicting the future energy consumption rate of nodes is important because accurate prediction helps determine the energy requirement to ensure the nodes’ survival until the next replenishment. Consequently, the failure rate of critical nodes in the network can be reduced, leading to a decrease in the EMR. Therefore, the accuracy of node energy consumption rate prediction is negatively correlated with the EMR.
- Based on the energy consumption rate prediction of the nodes, a charging scheduling scheme is developed to meets the Dual Constraints of Nodes’ energy requirements and MC’s capability (DCNM problem).

Thus, the issue is formally described as

$$Max\;\left(\sum_{i=1}^{N}\left(f\left({\vec{p_{T}\left(s_{i}\right)}}_{x}\right)\;\left|\;x\in \left[1,2^{K}\right]\right.\right)\right)\;s.t.\;(1),\;(4)\;\mathrm{and}\;(5)$$

The symbols mentioned in this section and their definitions are shown in Table 1.

## 4. Construction of Energy Consumption Balanced Tree

In order to minimize the load difference among nodes and thereby reduce node failure rate, we first construct an Energy Consumption Balanced Tree (ECBT) that includes all nodes in the network. The BS serves as the root.

### 4.1. Node Energy Consumption Model for Sending and Receiving Data

Without a loss of generality, the energy consumption model proposed by W. R. Heinzelman [35] is adopted in our method. Thus, for a sensor node, to transmit a 1-bit message to its parent, the energy consumption of it is

(8) $$\left\{\begin{array}{l} p^{t}\left(s_{i},s_{p}\right)=e_{elec}+\varepsilon_{fs}\cdot d^{2}\left(s_{i},s_{p}\right)\;\left|\;d\left(s_{i},s_{p}\right)<d_{0}\right.\\ p^{t}\left(s_{i},s_{p}\right)=e_{elec}+\varepsilon_{amp}\cdot d^{4}\left(s_{i},s_{p}\right)\left|\;d\left(s_{i},s_{p}\right)\geq d_{0}\right. \end{array}\right.$$

and to receive this message, the radio expends

(9) $$p^{r}\left(s_{i}\right)=e_{elec}$$

This model shows the communication process between *s_i_* and its parent, i.e., *s_p_*, and the parameter *d*(*s_i_*, *s_p_*) represents the distance between them. When *d*(*s_i_*, *s_p_*) < *d*_0_, the model can be regarded as a “free space environment” (low signal attenuation), otherwise it is a “multi-path fading environment” (large signal attenuation). *d*_0_ denotes the threshold distance and its value is (*ε_fs_*/*ε_amp_*)^1/2^. Moreover, *P^t^*(*s_i_*, *s_p_*) and *P^r^*(*s_i_*) are the energy consumption rate of *s_i_* during its sending and receiving phase, respectively. Meanwhile, *e_elec_* is the unit energy consumption of the circuit. *ε_fs_* and *ε_amp_* are the constant parameters of the signal amplifier in the free space and multi-path fading environment.

The energy consumption of node *s_i_* in a round of service time *T* is considered to be its workload. In the worst case, there is a constant stream of events at all of the POIs within the range *R*(*s_i_*) over *T*. Thus, the maximum energy consumption of *s_i_* during this period can be expressed as

(10) $$E^{T}\left(s_{i}\right)=\underset{POI_{l}\in R\left(s_{i}\right)}{\left|POI_{l}\right|}\cdot m\cdot \left(p^{r}\left(s_{i}\right)+p^{t}\left(s_{i},s_{p}\right)\right)$$

In this formula, |POI*_l_*| represents the number of POIs that *s_i_* can monitor, and *m* represents the size of data generated by a node sensing a POI during *T*.

### 4.2. Energy Consumption Balanced Tree Construction (ECBTC) Based on Greedy Strategy

It is well known that in multi-hop transmission WRSNs, the nodes located close to the BS consume a significant amount of energy since they usually forward a large volume of data. As the root nodes of huge subtrees, their importance in the network is self-evident. Hence, the primary objective of constructing an ECBT is to balance the load among subtrees rather than individual nodes. In this way, the survival rate of the nodes can be significantly enhanced while reducing the EMR. To achieve this goal, it is necessary to ensure that the load difference among the subtrees of the ECBT is as consistent as possible before and after any node joins. Obviously, this is an NP-hard problem. To this end, we propose the following greedy strategy.

Step 1. Assume the BS as the root and generate the initial ECBT (line 1).

**Figure 2:**
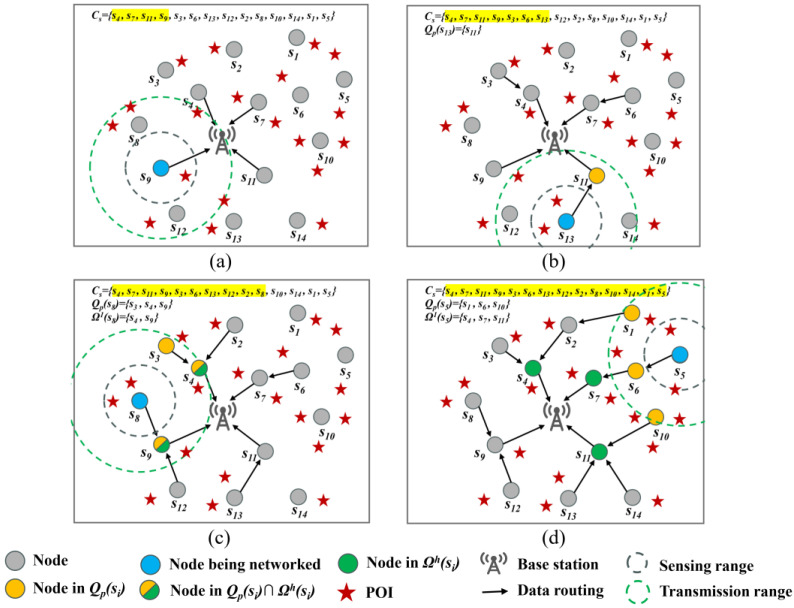
An example of ECBTC; (**a**–**d**) are four snapshots of the process.

Step 2. The nodes in the network are ranked in descending order of their Euclidean distance from the BS to create the queue *C_s_* (line 3). For instance, in Figure 2, *C_s_* = {*s*_4_, *s*_7_, *s*_11_, *s*_9_, *s*_3_, *s*_6_, *s*_13_, *s*_12_, *s*_2_, *s*_8_, *s*_10_, *s*_14_, *s*_1_, *s*_5_}. The highlighted nodes are those that have already been added to the tree.

Step 3. If *C_s_* = *Φ*, the construction process of ECBT ends. Otherwise, we investigate the first node in *C_s_* (denoted as *s_i_*). If the BS is its neighbor, *s_i_* is directly added to the ECBT with the BS as its parent (e.g., *s*_4_, *s*_7_, *s*_11_, and *s*_9_ in Figure 2a). Following that, *s_i_* is removed from *C_s_* and Step 3 is performed again (line 5–9). Otherwise, the algorithm jumps to Step 4. In this way, both the transmission distance from the node to the BS can be shortened and the number of children of the BS can be increased as much as possible, so as to share the workload.

Step 4. *Q_p_*(*s_i_*) is defined as the set of nodes that have previously been in the ECBT among all of the neighbors of *s_i_*, which is also the set of candidate parents. If there is only one node in *Q_p_*(*s_i_*), it will be considered as the parent of *s_i_*. Therefore, *s_i_* is added to the ECBT (e.g., in Figure 2b, *s*_13_ selects *s*_11_ as its parent and is consequently added to the tree because *s*_11_ is the only node in the ECBT among all of the neighbors of *s*_13_). Subsequently, *s_i_* is removed from *C_s_* and the algorithm moves to Step 3 again (line 27–28). Otherwise, Step 5 is carried out.

Step 5. Without a loss of generality, we suppose that the nodes in *Q_p_*(*s_i_*) belong to a number of subtrees rooted at the nodes that are *h* hops away from the BS (marked as *s_r_^h^*), respectively. Here, the set of these root nodes is denoted as Ω*^h^*(*s_i_*). The initial value of *h* is 1, i.e., the subtrees rooted at the children of the BS are investigated first (line 15–23).

- If *Q_p_*(*s_i_*) ∩ Ω*^h^*(*s_i_*) ≠ *Φ*, the node closest to *s_i_* in this intersection set is selected as its parent. Subsequently, we remove *s_i_* from *C_s_* and the algorithm jumps to Step 3. For example, in the given scenario depicted in Figure 2c, *h* = 1 and there are two nodes *s*_4_ and *s*_9_ in *Q_p_*(*s*_8_) = {*s*_3_, *s*_4_, *s*_9_} which are also the roots of the current subtrees (i.e., belong to Ω^1^(*s*_8_)). As a result, *s*_8_ takes the closer node *s*_9_ as its parent. The purpose of this is to decrease the number of hops between *s_i_* and the BS and to shorten its single-hop transmission distance as much as possible in order to minimize the value of *p^t^*(*s_i_*, *s_p_*).
- If *Q_p_*(*s_i_*) ∩ Ω*^h^*(*s_i_*) = *Φ*, the algorithm jumps to Step 6.

Step 6. Find out whether Formula (11) holds for each node in Ω*^h^*(*s_i_*):

(11) $$E^{T}\left(s_{r}^{h}\right)+\underset{POI_{l}\in R\left(s_{i}\right)}{\left|POI_{l}\right|}\cdot m\cdot \left(p^{r}\left(s_{r}^{h}\right)+p^{t}\left(s_{r}^{h},s_{p}^{h-1}\right)\right)<\alpha \cdot \left(\sum_{s_{r}^{h}\in \Omega^{h}\left(s_{i}\right)}E^{T}\left(s_{r}^{h}\right)/\left|\Omega^{h}\left(s_{i}\right)\right|\right)$$

Among them, *s_p_^h^*^−1^ is the parent of *s_r_^h^*. The parameter *α* is adjustable with a value between 1 and 2, and can be set according to the specific network situations. The function of Formula (11) is to determine if when *s_i_* is added to the subtree with a node as the root (denoted as *s_r_^h^*), the overall energy consumption of *s_r_^h^* in a round of service time is less than the average energy consumption of all of the root nodes in Ω*^h^*(*s_i_*) before *s_i_* is added.

If there is a node *s_r_^h^* that makes Formula (11) invalid, it indicates that adding *s_i_* to the subtree with *s_r_^h^* as the root will potentially increase the imbalance of energy consumption among the subtrees. Therefore, all of the candidate parents of *s_i_* that exist in the subtree rooted at the *s_r_^h^* are removed from *Q_p_*(*s_i_*) (line 18–20) (e.g., *s*_1_, *s*_10_ in Figure 2d). Otherwise, no operation is performed.

Step 7. If *Q_p_*(*s_i_*) is empty at this moment, then we roll back the “remove” operation in Step 6. Next, the node closest to *s_i_* in *Q_p_*(*s_i_*) is selected as its parent, and then we remove *s_i_* from *C_s_*, and the algorithm returns to Step 3. If there is only one node in *Q_p_*(*s_i_*) at this time, it is chosen as the parent of *s_i_*. Then we remove *s_i_* from *C_s_*, and the algorithm jumps to Step 3 (line 24–33). Otherwise, after making *h* = *h* + 1, Step 5 is carried out again.

An example of ECBTC and its pseudo-code are shown in Figure 2 and Algorithm 1, respectively. The parameters mentioned in this section and their definitions are shown in Table 2.
**Algorithm 1.** Energy Consumption Balanced Tree Construction, ECBTC**Input:** All nodes and POIs of the network. **Output:** ECBT *Ω*.  1:*Ω* = *s_BS_*, *C_E_* = {*E^T^*(*s*_1_), *E^T^*(*s*_2_),…, *E^T^*(*s_N_*)};  2:**for** *E^T^*(*s_i_*) = 0 **in** *C_E_*;  3:*C_s_* ← sort all nodes by distance (*s_i_*, *s_BS_*);  4:**for** *s_i_* **in** *C_s_***:**  5:   **if** *s_BS_* is the neighbor of *s_i_***:**  6:      connect (*s_BS_*, *s_i_*);  7:      update *E^T^*(*s_i_*) of *s_i_*;  8:      **continue**;  9:   **end if**;10:   *Q_p_*(*s_i_*) = *Φ*;
11:   **for** *s_j < i_* **in** *C_s_***:**12:      **if** *s_j_* is the neighbor of *s_i_***:**13:         add *s_j_* to *Q_p_*(*s_i_*);14:   **end for**;15:   *h* = 1;16:   **while** size (*Q_p_*(*s_i_*)) > 1 && *Q_p_*(*s_i_*)*∩Ω^h^*(*s_i_*) == *Φ* **do:**17:      **for** *s_r_^h^* **in** *Ω^h^*(*s_i_*)**:**18:         **if** *s_r_^h^* does not satisfy Equation (11)**:**19:            **remove** *s_p_* from *Q_p_*(*s_i_*) which belong to the sub-trees of *s_r_^h^*;20:         **end if**;21:      **end for**;22:      *h*++;23:   **end while**;24:   **if** *Q_p_*(*s_i_*) == *Φ***:**25:      roll back last remove operation;26:   **end if**;27:   **if** size(*Q_p_*(*s_i_*)) == 1**:**28:      get *s_p_* from *Q_p_*(*s_i_*);29:   **else:**30:      get *s_p_* of the shortest distance(*s_p_*, *s_i_*) from *Q_p_*(*s_i_*)∩Ω*^h^*(*s_i_*);31:   **end if**;32:   connect(*s_p_*, *s_i_*);33:   update *E^T^*(*s_r_*) of *s_r_* in path(*s_BS_*, *s_i_*);34:**end for**;35:**return** Ω;

## 5. Energy Consumption Rate Prediction for Nodes

Now, the energy consumption of the nodes in the same layer of ECBT has been basically balanced. To address the MEECR problem, we further aim to predict the energy consumption rates of nodes in the future duration, i.e., *T*. Thus, the amount of energy that the MC needs to replenish each node can be estimated, so as to efficiently sustain the operations of nodes and reduce the EMR. Firstly, the value range of *T* is calculated out by considering the charging requirement of the nodes and the service capability of the MC. Then, based on the properties of Poisson processes, an evaluation function for the accuracy of the nodes’ energy consumption rate prediction, which can be adapted to the different number of POIs within *R*(*s_i_*), is constructed. Finally, we propose a method of the near-optimal solution for the energy consumption rate prediction oriented to the alternating knapsack problem.

### 5.1. The Value Range of T

As previously indicated, setting a fixed value of *T* for the MC to traverse nodes not only regulates its charging behavior, but also enhances the accuracy of their energy consumption rate prediction. Obviously, the value of *T* cannot be too large because it should ensure that each replenished node can survive until the MC serves it again. That is, Formula (12) is as follows:

(12) $$T<\beta \cdot E_{s}/\underset{i\in \left[1,N\right]}{Max}\left(\omega_{2}\left(s_{i}\right)\right)$$

In Formula (12), *E_s_* is the full battery capacity of a node, $\left.Max\left(\omega_{2}\left(s_{i}\right)\right)\right|i\in \left[1,N\right]$ represents the maximum value of the energy consumption rate of all nodes in the network, and *β* is an adjustable parameter with a value between 0 and 1. Thus, it can be seen that once the network topology is established, this upper limit value is fixed.

On the other hand, the value of *T* should not be too small. It is crucial to ensure that the MC has enough time to serve all nodes under its energy constraint. Hence, according to the aforementioned Formulas (3) and (5), we have the following:

(13) $$T\geq D_{j}/v+\sum_{s_{i}\in O_{j}}T^{c}\left(s_{i}\right)$$

The right side of Formula (13) is the lower limit of *T*. Nevertheless, the duration *T^c^*(*s_i_*) for which MC replenishes *s_i_* in the *j*-th round is currently unknown before MC starts charging scheduling (i.e., *T^c^*(*s_i_*) can be determined after obtaining the optimal solution of the energy consumption rate prediction of *s_i_*). Therefore, an approximate method is adopted here to estimate the value of *T^c^*(*s_i_*) in order to get the lower limit value of *T*. To simplify the explanation, *T^c^*(*s_i_*) is denoted as *T_h_^c^*(*s_i_*) in the following expression, where *h* is the hop counts between *s_i_* and the BS.

Here, we consider the worst case that all nodes that are one-hop away from the BS are replenished by the MC only when they are exhausted from the full energy *E_s_* to zero, without being served by the MC during this period. Thus, it is easy to know

(14) $$T_{1}^{c}\left(s_{i}\right)=E_{s}/\left(\eta \cdot p^{c}\right)$$

We assume that *C_h_* represents the set of all nodes in the network that are *h* hops away from the BS, and the number of nodes in the set is |*C_h_*|. The mean Euclidean distance from nodes in *C_h_* to the BS is denoted as $\bar{d_{h}}$, and *p^t^*(*d*) represents the power consumption of a node that transmits data to a distance of *d* meters. Then it is evident that $\left(\left|C_{1}\right|\cdot E_{s}\right)/\left(e_{elec}+p^{t}\left(\bar{d_{1}}\right)\right)$ is the size of the data packet uploaded to the BS by all of the nodes that are one-hop away from the BS in the worst case described above. $1-\left(\sum_{j=1}^{h-1}\left|C_{j}\right|/N\right)$ denotes the proportion of nodes whose hop counts to the BS are no less than *h*. Furthermore, $e_{elec}+p^{t}\left(\bar{d_{h}}-\bar{d_{h-1}}\right)$ represents the energy consumption of the *h*th-hop node for uploading 1 bit of data to its parent (i.e., the (*h* − 1)th hop node). Therefore, for any node *s_i_* which is *h* hops away from the BS (*h* > 1), the duration for the MC to charge it each time can be estimated by Formula (15).

(15) $$T_{h}^{c}\left(s_{i}\right)=\left(\left(\left|C_{1}\right|\cdot E_{s}\right)/\left(e_{elec}+p^{t}\left(\bar{d_{1}}\right)\right)\right)\cdot \left(1-\left(\sum_{j=1}^{h-1}\left|C_{j}\right|/N\right)\right)\cdot \left(e_{elec}+p^{t}\left(\bar{d_{h}}-\bar{d_{h-1}}\right)\right)/\left(\eta \cdot p^{c}\right)$$

It is easy to know that, based on the estimation according to Formula (15), the nodes with the same number of hops are assigned the same charging duration. As known from Section 4.2, the workload between the nodes above has been basically balanced with the help of ECBTC. As a result, the estimation is reasonable.

### 5.2. The Evaluation Function f(.) for Energy Consumption Rate Prediction

As previously stated, in MEECR, the purpose of setting evaluation function $\left.f\left({\vec{p_{T}\left(s_{i}\right)}}_{x}\right)\;\right|\;x\in \left[1,\;2^{K}\right]$ is to acquire a quantitative index $f\left({\vec{p_{T}\left(s_{i}\right)}}_{x}\right)$ for every candidate solution ${\vec{p_{T}\left(s_{i}\right)}}_{x}$ in the energy consumption rate vector solution space *C*(*s_i_*) of *s_i_*. The larger the index is, the better the candidate solution is, i.e., using this solution to predict the energy consumption rate of *s_i_* within a future *T* has the best effect. To this end, we establish the correlation between sensing events and energy consumption rate based on the property of the Poisson process. Afterwards, it is classified according to the number of POIs that can be monitored by a node.

#### 5.2.1. Only One POI within *R*(*s_i_*)

We assumed in the previous section that the arrival process of the events on each POI*_l_* obeys a Poisson process with intensity *λ_l_*. Accordingly, the probability of *δ* events occurring at a POI*_l_* in time duration Δ*t* is

(16) $$P\left(X=\delta \right)={\left(\lambda_{l}\cdot \Delta t\right)}^{\delta }\cdot e^{-\lambda_{l}\cdot \Delta t}/\delta !$$

As mentioned before, there are only two values, i.e., *ω*_1_ and *ω*_2_(*s_i_*), for the energy consumption rate of any node *s_i_*. Therefore, in order to simplify the computational complexity, it is regulated here that if *s_i_* has the same value of *p_k_*(*s_i_*) in several consecutive time slices within a *T*, they will be merged into a “time interval”. Therefore, the “energy consumption rate vector of *s_i_* within a *T*” described in Formula (7) can also be expressed by Formula (17) as follows.

(17) pTsi→=p1si, τsi1, p2si, τsi2, …, pK′si, τK′si | K′≤K

Each two-tuple (*p_y_*(*s_i_*), *τ_y_*(*s_i_*))|*y*∈[1, *K*′] in Formula (17) shows that the energy consumption rate of *s_i_* in the time interval *τ_y_*(*s_i_*) is *p_y_*(*s_i_*). Therefore, it is easy to know that for any POI*_l_* monitored by *s_i_*, the average probability of events occurring can be expressed as

(18) $$\bar{P_{\omega_{2}\left(s_{i}\right)}\left(\delta \geq 1\right)}=\left(\sum_{y^{\prime }}P\left(\left.\delta \geq 1\;\right|\;\tau_{y^{\prime }}\left(s_{i}\right)\right)\cdot \tau_{y^{\prime }}\left(s_{i}\right)\right)/\sum_{y^{\prime }}\tau_{y^{\prime }}\left(s_{i}\right)$$

Among them, *τ_y_*_′_(*s_i_*) ∈ arg $\vec{p_{T}\left(s_{i}\right)}$ where *p*_*y*′_(*s_i_*) = *ω*_2_(*s_i_*). *P*(*δ* ≥ 1|*τ_y_*_′_(*s_i_*)) denotes the probability of events occurring within *τ_y_*_′_(*s_i_*). According to Formula (16), the above formula can be further derived as

(19) $$\begin{array}{ll} \bar{P_{\omega_{2}\left(s_{i}\right)}\left(\delta \geq 1\right)} & =\left(\sum_{y^{\prime }}\left(1-P\left(\left.\delta =0\;\right|\;\tau_{y^{\prime }}\left(s_{i}\right)\right)\right)\cdot \tau_{y^{\prime }}\left(s_{i}\right)\right)/\sum_{y^{\prime }}\tau_{y^{\prime }}\left(s_{i}\right)\\ & =\left(\sum_{y^{\prime }}\left(1-e^{-\lambda_{l}\cdot \tau_{y^{\prime }}\left(s_{i}\right)}\right)\cdot \tau_{y^{\prime }}\left(s_{i}\right)\right)/\sum_{y^{\prime }}\tau_{y^{\prime }}\left(s_{i}\right)\\ & =1-\sum_{y^{\prime }}e^{-\lambda_{l}\cdot \tau_{y^{\prime }}\left(s_{i}\right)}\cdot \tau_{y^{\prime }}\left(s_{i}\right)/\sum_{y^{\prime }}\tau_{y^{\prime }}\left(s_{i}\right) \end{array}$$

And for any POI*_l_* that is monitored by *s_i_*, the average probability of no event occurring can be expressed as

(20) $$\bar{P_{\omega_{1}}\left(\delta =0\right)}=\left(\sum_{y^{\prime \prime }}P\left(\left.\delta =0\;\right|\;\tau_{y^{\prime \prime }}\left(s_{i}\right)\right)\cdot \tau_{y^{\prime \prime }}\left(s_{i}\right)\right)/\sum_{y^{\prime \prime }}\tau_{y^{\prime \prime }}\left(s_{i}\right)$$

In Formula (20), *τ_y_*_″_(*s_i_*) ∈ arg $\vec{p_{T}\left(s_{i}\right)}$ where *p_y_*_″_(*s_i_*) = *ω*_1_, and *P*(*δ* = 0|*τ_y_*_″_(*s_i_*)) denotes the probability that no event occurs within *τ_y_*_″_(*s_i_*). Similarly, we have

(21) $$\bar{P_{\omega_{1}}\left(\delta =0\right)}=\left(\sum_{y^{\prime \prime }}e^{-\lambda_{l}\cdot \tau_{y^{\prime \prime }}\left(s_{i}\right)}\cdot \tau_{y^{\prime \prime }}\left(s_{i}\right)\right)/\sum_{y^{\prime \prime }}\tau_{y^{\prime \prime }}\left(s_{i}\right)$$

As a result, the evaluation function for the accuracy of the energy consumption rate prediction is characterized by Formula (22) as follows.

(22) $$f\left({\vec{p_{T}\left(s_{i}\right)}}_{x}\right)=\gamma \left(s_{i}\right)\cdot \bar{P_{\omega_{2}\left(s_{i}\right)}\left(\delta \geq 1\right)}+\left(1-\gamma \left(s_{i}\right)\right)\cdot \bar{P_{\omega_{1}}\left(\delta =0\right)}$$

Among them, *γ*(*s_i_*) is used to regulate the importance of the events monitored by *s_i_* and takes a value between 0 and 1.

#### 5.2.2. Multiple POIs within *R*(*s_i_*)

Given that the events occurring at different POIs are independent, the following two conditions are considered.

- For a number of POIs with a consistent value *λ_l_*, they can be regarded as a “fused POI”. Its Poisson intensity is considered to be the sum of the Poisson intensities of the POIs.
- For a number of POIs with different values of *λ_l_*, the evaluation function of the energy consumption rate prediction for *s_i_* is denoted as the sum of the evaluation functions of each single POI*_l_* within *R*(*s_i_*).

(23) $$f\left({\vec{p_{T}\left(s_{i}\right)}}_{x}\right)=\sum_{\left|POI_{l}\right|}f{\left({\vec{p_{T}\left(s_{i}\right)}}_{x}\right)}_{l}$$

### 5.3. Approximate Optimal Solution for Energy Consumption Rate Prediction

The function *f*(.) provides a specific quantitative index for predicting the energy consumption rate of nodes. Thus, to obtain an approximate optimal solution of *f*(.), respectively, the nodes are further classified into the following four categories based on different tasks they undertake.

#### 5.3.1. The Node Does Not Monitor Any POIs (i.e., *R*(*s_i_*) = *Φ*) and Has No Children

Obviously, according to the definition of this paper, the energy consumption rate of this type of node is always *ω*_1_. Therefore, there is no need to predict anything.

#### 5.3.2. The Node Monitors POIs (i.e., *R*(*s_i_*) = *Φ*), but Has No Children

As mentioned in Section 3.2, the solution space *C*(*s_i_*) still remains of size 2*^K^* even if we assign the energy consumption rate of *s_i_* to only two specific values, *ω*_1_ and *ω*_2_(*s_i_*). When the number of time slices *K* is large, the complexity of finding the optimal solution for $f\left({\vec{p_{T}\left(s_{i}\right)}}_{x}\right)$ is high. Thus, we transform the problem into a variant of the multiple knapsack problem. Then, a dynamic programming method is employed to get an approximate optimal solution. This method effectively reduces the time complexity to a polynomial level.

For each two-tuple (*p_y_*(*s_i_*), *τ_y_*(*s_i_*)) | *y* ∈ [1, *K*′] in ${\vec{p_{T}\left(s_{i}\right)}}_{x}$, the goal of MEECR is to determine the values of *p_y_*(*s_i_*) and *τ_y_*(*s_i_*) such that $f\left({\vec{p_{T}\left(s_{i}\right)}}_{x}\right)$ is maximal. Since $\sum_{y=1}^{K^{\prime }}\tau_{y}\left(s_{i}\right)=T$, this can be considered to be a knapsack problem. That is, the sum of the time intervals *T* represents the weight of the knapsack. The two-tuple (*p_y_*(*s_i_*), *τ_y_*(*s_i_*)) is the equivalent of the item with weight *τ_y_*(*s_i_*), and the value of the item is characterized by the evaluation function $f\left({\vec{p_{T}\left(s_{i}\right)}}_{x}\right)$. From the previous description, the energy consumption rate of *s_i_* in two adjacent time intervals (i.e., *τ_y_*_′_(*s_i_*) and *τ_y_*_″_(*s_i_*)) is different. If one of them is *ω*_1_, then the other is *ω*_2_(*s_i_*). That is, the items that are put into the backpack twice in a row must be different. Hence, we call it the “alternating backpack problem”.

It is evident that *τ_y_*(*s_i_*) can take on any value inside the set {*T*/*K*, 2*T*/*K*,…, *T*}, so there are 2*K* types of items in total. In addition, the number of the items weighting *τ_y_*(*s_i_*) (denoted as *m_y_*) needs to satisfy

(24) $$m_{y}\cdot \tau_{y}\left(s_{i}\right)\leq T$$

According to the property of “alternating backpack” described above, the maximum value of *m_y_* can be calculated out by Formula (25).

(25) $$m_{y}=\left\lfloor T/\left(\tau_{y}\left(s_{i}\right)+T/K\right)\right\rfloor +\left\lfloor T\mathrm{mod}\left(\tau_{y}\left(s_{i}\right)+T/K\right)/\tau_{y}\left(s_{i}\right)\right\rfloor$$

For clarity, an example is shown in Table 3. Assume *T* = 5 h and *K* = 5. Therefore, there are 2*K* = 10 types of items in total. The diagrams in the table are the backpacks that are filled with the maximum number of current items. In particular, the energy consumption rate of *s_i_* during the blue and red time intervals are *ω*_1_ and *ω*_2_(*s_i_*), respectively. For instance, the time interval *τ_y_*(*s_i_*) of the first item is one hour, and the energy consumption rate *p_y_*(*s_i_*) is *ω*_1_. It is obvious that under the constraint of “alternating backpacks”, the maximum number of items will be three (i.e., three blue time intervals in that diagram) in this case. As an additional example, the time interval *τ_y_*(*s_i_*) of the fourth item is two hours, and the energy consumption rate *p_y_*(*s_i_*) is *ω*_2_(*s_i_*). It is easy to know that the maximum number of items is two (i.e., two red time intervals of two hours in the diagram) in this case, as any more would not satisfy the constraint of “alternating backpacks”.

Thus, a dynamic programming approach is used to solve the alternating knapsack problem. Firstly, the 2*T* types of items are expanded by rows (similar to converting a multi-knapsack problem into a 0–1 knapsack problem), so that each item type has a quantity of one. Then, the attributes of each item are defined as (*ID*, *τ_ID_*, *p_ID_*), where *τ_ID_* and *p_ID_* are the weight and value of the item, respectively. Furthermore, the state *dp*(*ID*, *χ*, *p*) represents the maximum profit when there are *ID* items available for selection. The knapsack capacity is *χ* and the last placed item’s *p_ID_* ∈ {*ω*_1_, *ω*_2_(*s_i_*)}. As a result, the state transition equation is shown as follows.

(26) $$dp\left(ID,\chi ,p\right)=\left\{\begin{array}{l} \max\left(dp\left(ID-1,\chi ,\omega_{1}\right),\;dp\left(ID-1,\chi -\tau_{ID},\omega_{2}\left(s_{i}\right)\right)+p_{ID}\right)\;|\;p=\omega_{1}\\ \max\left(dp\left(ID-1,\chi ,\omega_{2}\left(s_{i}\right)\right),\;dp\left(ID-1,\chi -\tau_{ID},\omega_{1}\right)+p_{ID}\right)\;|\;p=\omega_{2}\left(s_{i}\right) \end{array}\right.$$

Now, it is possible to find an approximate optimal solution $\vec{p_{T}\left(s_{i}\right)}$ for the energy consumption rate prediction of *s_i_* in polynomial time. The pseudo-code is shown in Algorithm 2.
**Algorithm 2.** Solution for the alternating knapsack problem**Input:** Evaluation function *f*(·), the node *s_i_*, the POI*_l_* monitored by *s_i_*, the number of time slices *K*, and schedule cycle *T*. **Output:** Energy cost prediction $\vec{p_{T}\left(s_{i}\right)}$ of *s_i_*.
  1:goods = *Φ*, *ID* = 1;  2:**for** *k* = 1 **to** *K***:**  3:   *τ_y_*(*s_i_*) = (*k* × *T*)/*K*;  4:   *m_y_* ← calculated by Equation (25);  5:   **for** *r* = 1 **to** *m_y_***:**  6:      add (*ID*++, *τ*_*y*″_(*s_i_*), *ω*_1_) to goods;  7:      add (*ID*++, *τ*_*y*′_(*s_i_*), *ω*_2_(*s_i_*)) to goods;  8:   **end for**;  9:**end for**;10:initialize *dp*[size(goods)][*T*][2] and let the first row and the first column be 0.11:traverse array *dp* row by row, filling it according to the state transition Equation (26).12:$\vec{p_{T}\left(s_{i}\right)}$ ← yield the approximate solution by backtracking *dp*;13:**return** $\vec{p_{T}\left(s_{i}\right)}$;

If |POI*_l_*| > 1, the following two cases can be discussed.

- The Poisson intensity of the events monitored by these POIs are the same (might as well all be denoted as *λ_l_*). As mentioned earlier, the occurrences of the events at different POIs are independent, so these POIs can be regarded as one “fused POI” and the Poisson intensity of it is |POI*_l_*|∙*λ_l_*.
- The Poisson intensity of events monitored by these POIs are different. Thus, an optimal solution ${\vec{p_{T}\left(s_{i}\right)}}^{l}$ can be found for each POI*_l_* by Algorithm 2. Subsequently, the final energy consumption rate prediction of *s_i_* is obtained by performing the merge operation, which is shown as follows.

(27) $$\vec{p_{T}\left(s_{i}\right)}=\cup_{l=1}^{\left|POI_{l}\right|}{\vec{p_{T}\left(s_{i}\right)}}^{l}$$

The informal description of this formula is that, for any time slice, the energy consumption rate of $\vec{p_{T}\left(s_{i}\right)}$ is *ω*_2_(*s_i_*) if there is a ${\vec{p_{T}\left(s_{i}\right)}}^{l}$ with an energy consumption rate of *ω*_2_(*s_i_*). Otherwise, the energy consumption rate of $\vec{p_{T}\left(s_{i}\right)}$ is *ω*_1_.

#### 5.3.3. The Node Does Not Monitor Any POIs (i.e., *R*(*s_i_*) = *Φ*), but Has Children

For this type of node *s_i_*, its energy consumption rate prediction can be considered as the result of the merge operation of the energy consumption rate prediction of its children (remarked as *s_j_*, and it assumes the set of all *s_j_* to be *DCN*(*s_i_*)). That is,

(28) $$\vec{p_{T}\left(s_{i}\right)}=\cup_{j=1}^{\left|DCN\left(s_{i}\right)\right|}{\vec{p_{T}\left(s_{i}\right)}}^{j}$$

The informal description of the above formula is similar to Formula (27).

#### 5.3.4. The Node Monitors POIs (i.e., R(s_i_) ≠ Φ) and Has Children

From Section 5.3.2 and Section 5.3.3 described above, it is easy to know that the final optimal solution for predicting the energy consumption rate of *s_i_* in this case can be obtained according to Formula (29).

(29) $$\vec{p_{T}\left(s_{i}\right)}=\left(\cup_{l=1}^{\left|POI_{l}\right|}{\vec{p_{T}\left(s_{i}\right)}}^{l}\right)\cup \left(\cup_{j=1}^{\left|DCN\left(s_{i}\right)\right|}{\vec{p_{T}\left(s_{i}\right)}}^{j}\right)$$

It should be noted that, to ensure the accuracy of the results from the merge operation, we make the following assumption. For each time slice with the same *k* in ${\vec{p_{T}\left(s_{i}\right)}}^{l}$ and ${\vec{p_{T}\left(s_{i}\right)}}^{j}$, if the values of *p_k_*(*s_i_*) are all *ω*_1_, the smallest probability value among them is selected as the final predicted probability of the *k-*th time slice of *s_i_*. Otherwise, the maximum probability value among the time slices whose *p_k_*(*s_i_*) are *ω*_2_(*s_i_*) is selected as the final predicted result for the *k*th time slice of *s_i_*. Thus, the optimal solution $\vec{p_{T}\left(s_{i}\right)}$ for the energy consumption rate prediction for *s_i_* can be finally expressed as follows.

(30) pTsi→=p1si, Psi1, p2si, Psi2, pksi, Psik, …, pKsi, PKsi

Here, *P_k_*(*s_i_*) is the probability that the energy consumption rate of *s_i_* is *p_k_*(*s_i_*) at the *k*th time slice.

So far, we have solved the MEECR problem. The pseudo-code corresponding to the above process is shown in Algorithm 3, and the parameters mentioned in this section and their definitions are shown in Table 4.
**Algorithm 3.** Solution of MEECR problem**Input:** The set of nodes *C_s_*, evaluation function *f*(·), the number of time slices *K*, and schedule duration *T*. **Output:** The solution *X*.
  1:*X* = *Φ*;  2:*C_s_* ← get the inverse order of *C_s_*;  3:**for** *s_i_* **in** *C_s_***:**  4:   initialize $\vec{p_{T}\left(s_{i}\right)}$ of *s_i_* and let every *P_k_*(*s_i_*) = 1 and *p_k_*(*s_i_*) = *ω*_1_;  5:   **for** POI*_l_* **in** *R*(*s_i_*)**:**  6:      ${\vec{p_{T}\left(s_{i}\right)}}^{l}$ ← apply Algorithm 2 to get the energy consumption rate prediction;  7:      $\vec{p_{T}\left(s_{i}\right)}$ = $\vec{p_{T}\left(s_{i}\right)}$ ∪ ${\vec{p_{T}\left(s_{i}\right)}}^{l}$;  8:   **end for**;  9:   **for** *s_j_* **in** *DCN*(*s_i_*)**:**10:      $\vec{p_{T}\left(s_{i}\right)}$ = $\vec{p_{T}\left(s_{i}\right)}$ ∪ $\vec{p_{T}\left(s_{j}\right)}$;11:   **end for**;12:   add $\vec{p_{T}\left(s_{i}\right)}$ to *X*;13:**end for**;14:**return** *X*;

## 6. Charging Scheduling Scheme Consistent with the MC’s Service Capability and Nodes’ Energy Requirement

After obtaining the near-optimal solution for the energy consumption rate prediction of each node, we construct the traversal path for MC during a round of service time. To address the DCNM problem, an iterative adjustment approach is proposed for the energy consumption rate prediction to ensure the MC’s service capability is consistent with the energy replenishment requirement of the nodes.

Here, we introduce a “hop count based multi-Hamiltonian traversal paths construction” method.

Step 1. At the beginning of a *T*, the MC departs from the BS, setting *h* = 1. Then, the node within *C_h_* which is closest to the BS is selected out as the first energy-replenishing target. Next, the shortest Hamiltonian path that traverses all nodes in *C_h_* from this node is constructed.

Step 2. Afterward, the MC moves along this path and serves each node in *C_h_* sequentially. When finishing charging the last node (denoted as *s_i_*), it checks whether the current value of *h* has reached the maximum number of layers of the ECBT. If so, the charging process of this round ends, and then the MC returns to the BS. Otherwise, after making *h* = *h* + 1, the algorithm goes to Step 3.

Step 3. The MC searches for the node within *C_h_* that is closest to *s_i_* (denoted as *s_j_*), and sets it as the starting point. Then, the shortest Hamiltonian path that traverses all of the nodes in *C_h_* is constructed. Next, the MC moves from *s_i_* to *s_j_*. After that, the algorithm goes to Step 2.

It is easy to find that the above “inside-out” charging mode effectively reduces the computational complexity by forming multiple traversal paths in a round of service time. Furthermore, it ensures that the MC gives higher priority to charge nodes with smaller hop counts from the BS.

**Figure 3:**
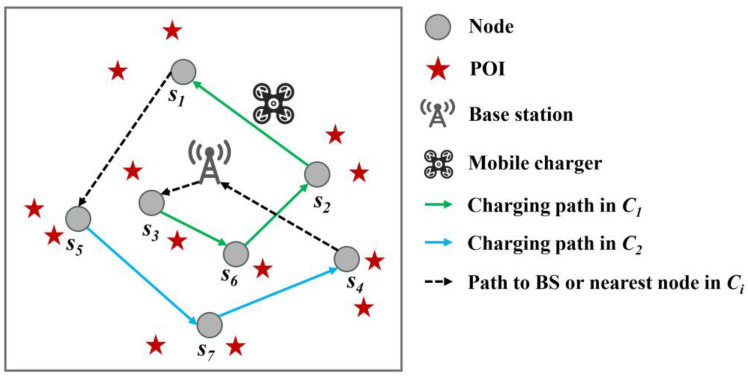
Multi-Hamiltonian path traversal mode based on hop count.

As shown in Figure 3, we suppose there are seven nodes in the network, with *C*_1_ = {*s*_1_, *s*_2_, *s*_3_, *s*_6_} and *C*_2_ = {*s*_4_, *s*_5_, *s*_7_}. The MC initially selects *s*_3_, which is closest to its current location (i.e., the location of the BS), as its first service object for this round. Subsequently, the first Hamiltonian path *s*_3_→*s*_6_→*s*_2_→*s*_1_, illustrated by the green solid line in this figure, is established. After sequentially charging these four nodes, MC selects *s*_5_, which is nearest to *s*_1_, as the next node to replenish energy. Furthermore, it establishes another Hamiltonian path *s*_5_→*s*_7_→*s*_4_ with *s*_5_ as the starting point, shown by the blue solid line in Figure 3. At last, after charging these three nodes in turn, the MC returns to the BS and completes a round of replenishment.

Obviously, the above traversal paths are almost unchanged once they are established (except when a node is dead, and a new Hamiltonian path containing all other nodes in *C_h_* is constructed according to Step 3). Thus, the value of *D_j_* in Formulas (2) and (3) can be calculated out now, and the value of *T^c^*(*s_i_*) can also be estimated through $\vec{p_{T}\left(s_{i}\right)}$. Therefore, we further check whether Formulas (4) and (5) hold. If they are valid, the MC can start a round of charging scheduling. Alternatively, it indicates that its service capability temporarily cannot meet the requirement of all nodes. Hence, the energy consumption rate prediction $\vec{p_{T}\left(s_{i}\right)}$ is adjusted iteratively to appropriately reduce the amount of the energy replenishment of some nodes, so as to ensure that the service capacity of the MC is as consistent as possible with the energy requirement of the nodes. The execution process is shown as follows.

(1) The time slice with the smallest value of *P_k_*(*s_i_*) among the time slices where *p_k_*(*s_i_*) is *ω*_2_(*s_i_*) in all nodes is selected. Then, the values of *p_k_*(*s_i_*) and *P_k_*(*s_i_*) are set to *ω*_1_ and 1 − *P_k_*(*s_i_*), respectively, i.e., the value of $\vec{p_{T}\left(s_{i}\right)}$ is fine-tuned.
(2) According to the fine-tuned $\vec{p_{T}\left(s_{i}\right)}$, we recheck the validity of Formulas (4) and (5). If they are valid, the adjustment process ends, and then the MC begins a round of replenishment. Otherwise, it jumps to step (1).

We now have a complete solution for the DCNM problem. The corresponding pseudo-code is shown in Algorithm 4.
**Algorithm 4.** Solution for the DCNM problem.**Input:** The set of nodes *C_s_*, the solution *X*, and battery capacity *E_MC_* of the MC. **Output:** The solution *Y*.
  1:*C_h_* = {*C*_1_, *C*_2_, …, *C_Ʊ_*} ← divide *C_s_* by hop count;  2:*Y* = *Φ*, *D* = *Φ*, *E* = *Φ*;  3:**for** *C_i_* **in** *C_h_***:**  4:   *H_i_* ← construct the Hamiltonian path of *C_i_*;  5:   *s_i_^start^* ← get *s_j_* of the shortest distance (*s_i_*_−1_*^end^*, *s_j_*) in *C_i_*;  6:   *s_i_^end^* ← *s_i_^start^* + *H_i_*;  7:   add (*H_i_*, *s_i_^start^*, *s_i_^end^*) to *D*;  8:**end for**;  9:**for** $\vec{p_{T}\left(s_{i}\right)}$ **in** *X***:**10:   calculate *E^o^*(*s_i_*) of *s_i_* according to Equation (1) and add it to *E*;11:**end for**;12:**while** not satisfy Equations (4) and (5) **do:**13:   select the time slice with the minimum *P_k_*(*s_i_*) from *X* which *p_k_*(*s_i_*) == *ω*_2_(*s_i_*) and let *p_k_*(*s_i_*) = *ω*_1_, *P_k_*(*s_i_*) = 1 − *P_k_*(*s_i_*);14:   recalculate *E*^o^(*s_i_*) and update it in *E*;15:**end while**;16:*Y* = {*D*, *E*};17:**return** *Y*;

## 7. Simulation Results

In this section, simulations are conducted to show the advantages of our proposed method. To verify the advantages of ECBT, we employed a routing algorithm that selects the nearest neighbor (NN) as the parent as a baseline for comparison. During the network topology construction phase of NN, all nodes are sorted in ascending order of their distance to the BS. Then, each node is traversed, selecting the nearest neighbor (i.e., the nodes within communication range) with an established route as the parent. ECBT–MEMR combines the ECBT and MEMR which is our proposed charging scheduling method. Similarly, NN–MEMR integrates the NN and MEMR as a benchmark method. To validate the advantages of ECBT–MEMR in minimizing the EMR, we choose RAND and LL [31] as the baseline for comparison. In the RAND method, the MC charges all nodes in a random sequence during each *T*. The LL method takes into account the dynamic changes of the nodes’ energy consumption rate and uses short-term prediction to develop the charging scheduling strategy, which is consistent with our proposed method.

We first explore the impact of parameter α (Formula (11)) in ECBTC on the EMR. Next, the performance of ECBT–MEMR and NN–MEMR under the different size of time slices and *T* is compared. Subsequently, we compare our method with two baselines (RAND and LL). The impact of the number of nodes and the maximum Poisson intensity of POI events on EMR are examined. Moreover, the node survival under different numbers of nodes is investigated. Finally, by randomly generating nodes’ locations, we verify the robustness of our method in different networks.

Parameter settings for the simulation are shown in Table 5. By default, the simulation uses the value of each parameter from the table unless stated otherwise.

### 7.1. Impact of Parameter α

**Figure 4:**
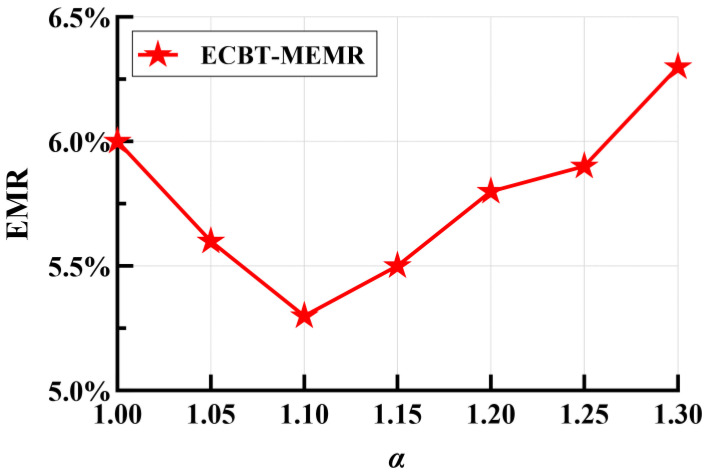
EMR vs. parameter *α*.

The parameter *α* reflects the energy consumption balance among nodes at the same layer in ECBT. The closer *α* is to 1, the more the smaller energy consumption differences are balanced. Figure 4 shows the impact of different values of *α* on the EMR when executing ECBT–MMDR. The overall trend indicates that as *α* increases, the EMR first decreases to about 5.3% (*α* = 1.1) and then increases steadily. Based on ECBTC, when determining whether each candidate parent is qualified to be a parent, the energy consumption balance is examined starting from the nodes with minimum *h*. When *α* is close to 1, even small energy consumption differences between nodes are considered unbalanced, leading to premature parent selection. Consequently, ECBTC might degrade into balancing energy consumption only among nodes with smaller *h*. This leads to an imbalance in the energy consumption of the remaining nodes, and may increase some nodes’ energy consumption due to selecting the more distant nodes as their parent. As mentioned in Section 6, the MC charges each node according to its hierarchical order of ECBT, starting from the BS. Consequently, the nodes with high energy consumption may die due to not being replenished in time, resulting in the occurrence of events missing.

As *α* increases, appropriate energy consumption differences are considered balanced. Thus, the energy consumption of the nodes with a larger value of *h* also gradually balances, thereby reducing the failure rate and EMR. When *α* increases to about 1.1, the EMR is at its lowest value, that is to say, the ECBTC effectively balances the energy consumption differences of the nodes with different *h*. Subsequently, as *α* continues to rise, excessive energy consumption differences are still considered to be balanced. Eventually, the algorithm degrades into NN–MMDR.

### 7.2. Impact of the Size of Time Slice

**Figure 5:**
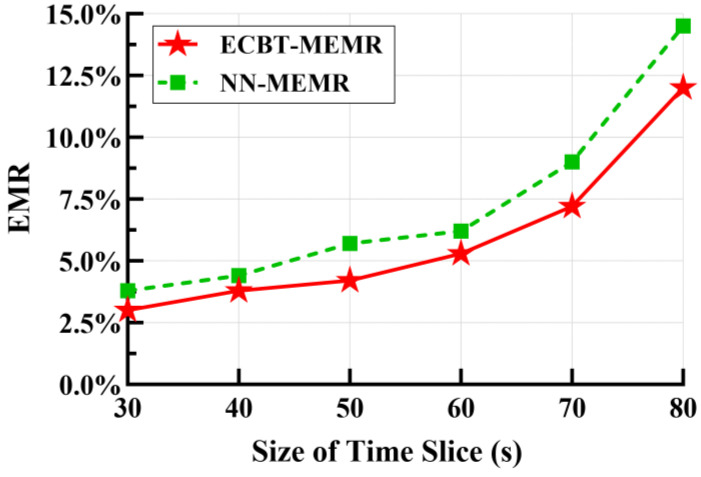
EMR vs. size of time slice.

For ECBT–MEMR and NN–MEMR, we examined the impact of different sizes of time slices on the EMR. As illustrated in Figure 5, for both of them, the EMR increases as the size of the time slice increases. When the size of the time slice is 30 s, the EMR is below 5%. But it exceeds 10% when the size of the time slice is 80 s and even reaches 14.5% under NN–MEMR. The main reason for this is that the accuracy of node energy consumption rate prediction depends on the size of the time slice and directly affects the result of the EMR. According to Section 5, the smaller the size of the time slice is, the more accurately the energy consumption rate variation in the nodes in a *T* can be depicted. Conversely, an excessively large time slice significantly reduces the accuracy of energy consumption rate prediction, increasing the probability of node death and thereby raising the EMR.

On the contrary, the smaller the time slice, the larger the solution space, which increases the time and space complexity of the algorithm. For ECBT–MEMR, it can be seen that reducing the size of the time slice from 60 s to 30 s decreases the EMR from 5.3% to 3.0%, but the solution space expands from 2^47^ to 2^94^. Thus, a balance between the EMR and the algorithm’s time–space overhead is required. Comparing ECBT–MEMR and NN–MEMR, it is evident that the former consistently outperforms the latter, primarily due to ECBT’s role in balancing energy consumption, thereby reducing the nodes’ significance and death probability. NN, on the other hand, only considers the transmission distance and ignores the energy consumption of the nodes, resulting in a higher node death probability. Furthermore, as the size of the time slice increases, the EMR in NN–MEMR rises more rapidly. When the time slice is 30 s, the EMRs of these two algorithms differ by 0.8%. But when the time slice is 80 s, they differ by 2.5%. This is because compared to ECBT–MEMR, NN–MEMR requires a higher accuracy of node energy consumption rate prediction. However, as analyzed earlier, a larger time slice leads to lower prediction accuracy, resulting in an incorrect assessment of the nodes’ energy requirement. At this point, nodes with higher energy consumption under NN–MEMR are more likely to die, which in turn increases the EMR.

### 7.3. Impact of the Scheduling Duration T

**Figure 6:**
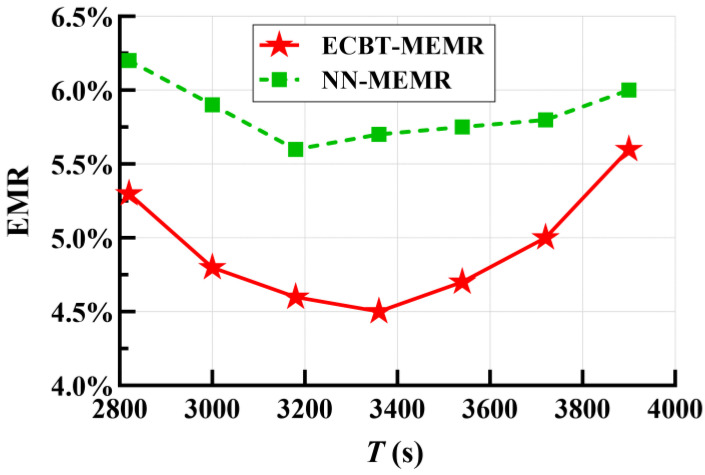
EMR vs. scheduling duration *T*.

According to the analysis mentioned in Section 5, we can get a range of [2814, 4028] for *T* with the help of some parameters in Table 3. To further examine the impact of its size on the EMR, we obtained the trend of the EMR with ECBT–MEMR and NN–MEMR through experiments, as shown in Figure 6. As *T* increases, the EMR first decreases, reaching an optimal value around *T* = 3420, and then gradually increases. According to Section 6, when *T* is too large or too small, the charging scheduling scheme adjusts the energy consumption rate predictions of some nodes (i.e., reduces the charging requirement of some nodes) to satisfy Formulas (4) and (5). Thus, it increases the probability of node death and consequently raises the EMR. In addition, for ECBT–MEMR, the average EMR is, overall, 0.92% lower than that of NN–MEMR, reflecting ECBT’s strong applicability under different sizes of *T*. However, it is important to note that as *T* (*T* > 3400) continues to increase, the EMR of ECBT–MEMR gradually approaches that of NN–MEMR. This is mainly because ECBT gradually fails to offset the performance differences due to the limited service capability of the MC.

### 7.4. Impact of the Number of Nodes

**Figure 7:**
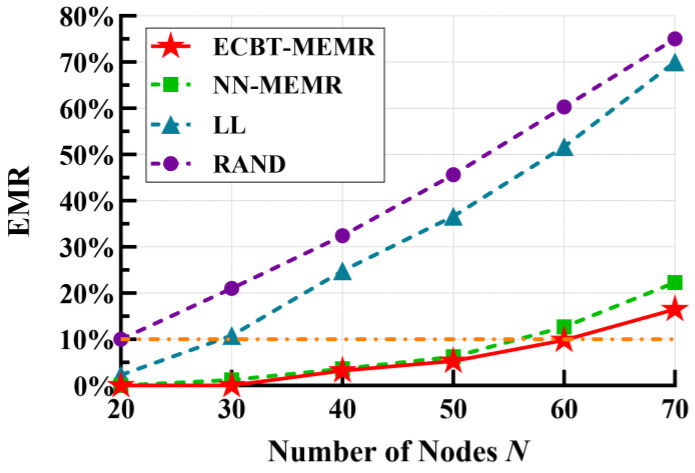
EMR vs. number of nodes.

Next, we compare the proposed method with LL and RAND to explore their performance under different numbers of nodes, i.e., *N*. Figure 7 shows the trend of the EMR for each method. As the value of *N* increases, the EMR also gradually increases, which is inevitable due to the limited service capability of the MC. For LL and RAND, some nodes die because they are not replenished in time. For NN–MEMR and ECBT–MEMR, some nodes die because the supplied energy is insufficient. However, with the same value of EMR = 10% (i.e., the orange reference line in the figure), the MC can serve about 40 and 30 more nodes by using ECBT–MEMR compared to RAND and LL, respectively. Additionally, compared to RAND, the values of the EMR in NN–MEMR and ECBT–MEMR reduce by 33.3% and 35.2% on average, respectively. Moreover, compared to LL, they reduce it by 25.0% and 26.9%, respectively. The advantages of our method become more apparent as the number of nodes increases. The main reason for the above results is that ECBT–MEMR fully considers the stochastic process of events occurring at the POI and predicts the energy consumption variations in the nodes over a *T* to determine the appropriate charging amount. When exceeding the MC’s service capability, the proposed method prioritizes the energy requirement of the nodes with a higher probability of events (i.e., higher energy consumption) to minimize the EMR. In contrast, LL simply uses the expectation of historical data as the short-term energy consumption rate of the nodes, lacking an accurate model of node energy consumption for the occurrence and disappearance of events, resulting in a lower accuracy of predictions. Additionally, LL is a request-based charging method in WRSNs. Nodes will die when too many charging requests emerge in a short time because the MC cannot reach them in time, regardless of whether or not they undertake critical tasks. RAND, on the other hand, does not consider the remaining energy of the nodes, resulting in the poorest performance. Furthermore, we also noticed that compared to ECBT–MEMR, the EMR of NN–MEMR is higher and the gap gradually increases as the number of nodes increases. This indicates that as the number increases, the balance of node energy consumption has a growing impact on the MC’s service quality.

**Figure 8:**
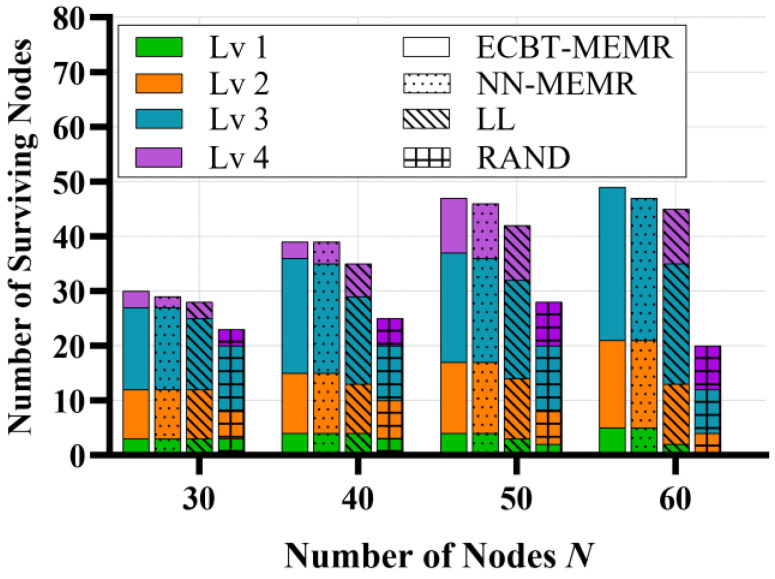
Number of surviving nodes.

To further demonstrate the superiority of our method, we show the number of surviving nodes in Figure 8. Different methods correspond to stacked bar charts with different fillings. The vertical axis represents the number of surviving nodes. In the experiment, the number of POIs monitored by each node and its offspring nodes is counted. Then the range of the number is categorized into four levels evenly, and thus obtains the level of each node (Lv1~Lv4). The higher the level is (e.g., Lv1 is higher than Lv2), the more POIs the node monitors. That is to say, the level indicates the importance of the node. We use bar charts of different colors to show the levels of surviving nodes. Overall, when the number of nodes does not exceed 50, both our method and LL can ensure more than 80% of nodes are alive. When the number of nodes reaches 60, only ECBT–MEMR maintains a node survival rate of approximately 81.6%, while all other methods fall below 80%. In addition, RAND performs the worst, leading to a gradual mass death of nodes. In addition, it is evident from the figure that the number of alive nodes in LL is not significantly less than our method, but their EMRs differ greatly. The reason is that, under the constraint of the MC’s service capability, ECBT–MEMR always prioritizes supplying sufficient energy to nodes that are more likely to undertake data tasks (i.e., nodes or their offspring nodes monitoring POIs where events are more likely to occur), while reducing the energy requirement of other nodes. In contrast, LL always strives to ensure every node is alive. Therefore, the death probability of each node is the same. When nodes undertaking important data tasks die, many events go missing. For instance, when the number of nodes is 60, although all nodes of Lv4 have died under the proposed method, nodes of Lv1 and Lv2 are still alive. This strategy allows many event reports from the POI to be successfully transmitted to the BS through high-level nodes, minimizing the EMR. In contrast, under LL, although some nodes of Lv4 are alive, some nodes of Lv1 and Lv2 have already died. It should be noted that once the high-level nodes die, even if their children remain alive, the data cannot be sent to the BS. This leads to the loss of many event reports and a rapid increase in the EMR.

### 7.5. Impact of the Maximum Value of Poisson Intensity

**Figure 9:**
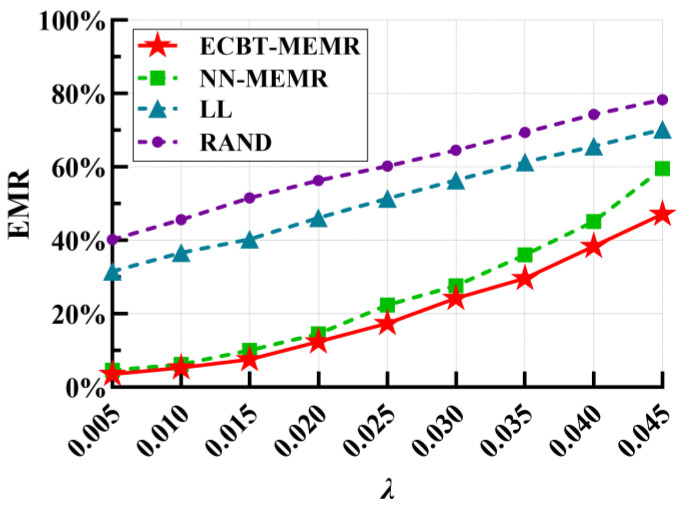
EMR vs. maximum poisson intensity *λ* of POI events.

Under the assumptions of ECBT–MEMR, the arrival of events at the POI follows a Poisson process. Hence, higher Poisson intensity results in more frequent events arriving. Figure 9 shows the impact of different maximum Poisson intensities on the EMR. It is apparent that as *λ* rises, the EMR also gradually increases. This is expected because the more frequently the events arrive, the more time slices are spent in a high energy consumption rate. Therefore, for LL and RAND, nodes are more likely to die because the MC cannot reach them in time. For our method, the primary reason is that the size of the time slice (the default value is 60 s) does not decrease with increases in *λ*, which means that a large time slice cannot accurately depict nodes’ energy consumption variations, significantly reducing prediction accuracy. For example, when *λ* is 0.04, the size of the time slice should be less than 1/0.04 = 25 s. Hence, this also explains why the EMR grows rapidly as *λ* increases in ECBT–MEMR and further confirms that the size of the time slice significantly affects the performance. Despite this, our method still outperforms the comparison methods, guaranteeing a lower EMR. Additionally, ECBT balances the energy consumption, which reduces the negative impact of the decreased accuracy of the energy consumption rate prediction, allowing ECBT–MEMR to further lower the EMR compared to NN–MEMR.

### 7.6. Performance Robustness

**Figure 10:**
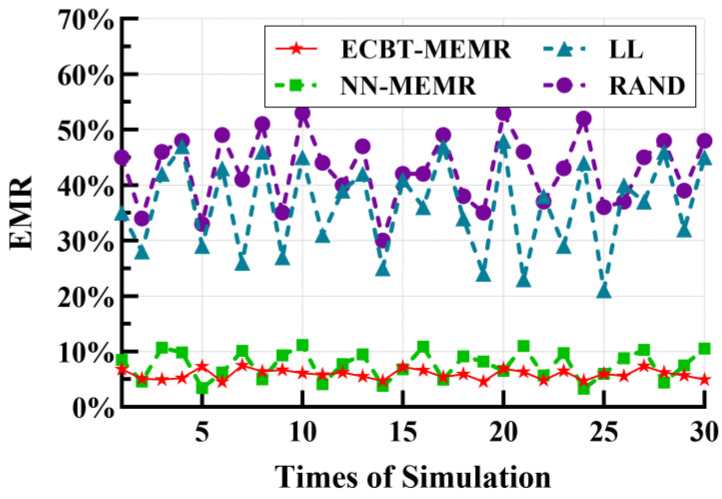
Robustness of the four methods.

Finally, we recorded the resulting EMR with each method in 30 independent simulation experiments. As shown in Figure 10, the EMR in LL and RAND are the most volatile, ranging from 21% to 53%. Their variances are 6.79 × 10^−3^ and 3.96 × 10^−3^, respectively. The primary reason for this is that these two algorithms exhibit significant randomness in charging scheduling and lack mechanisms to ensure the survival of nodes. When nodes are unevenly deployed in the network, it greatly increases the MC’s moving time and energy consumption, thereby raising the risk of node death, leading to a higher EMR. In contrast, ECBT–MEMR demonstrates stronger robustness with various node distributions, with variances of 0.65 × 10^−3^ for NN–MEMR and only 0.08 × 10^−3^ for ECBT–MEMR. This is due to the complete node survival mechanism. On the one hand, the energy consumption rate prediction is based on the network topology, which accurately evaluates nodes’ energy requirements. As a result, node survival is guaranteed across different network topologies. On the other hand, the periodic charging scheduling mode guarantees that nodes will be replenished after a *T*. Furthermore, ECBT–MEMR is more stable compared to NN–MEMR. When nodes are unevenly deployed, NN may accelerate the energy consumption rate of the nodes in some areas, whereas ECBT always strives to balance the energy consumption among nodes in the same layer, thereby reducing the probability of node death.

## 8. Conclusions and Future Work

In this study, we propose a charging scheduling method based on an event-oriented node energy consumption rate prediction to minimize the event missing rate (EMR). The distributional imbalance and temporal dynamics of node energy consumption rate are considered. Hence, an energy consumption balanced tree construction method is proposed to prolong the lifetime of each node. In order to evaluate the energy requirement of the nodes, we predict their energy consumption rate within a scheduling duration. Within it, the evaluation function for energy consumption rate prediction is explored. Subsequently, we transform the energy consumption rate prediction into a variant of the backpack problem, namely, the alternating backpack problem, and solve this by dynamic programming. After that, a charging scheduling scheme to satisfy the MC’s service capability and nodes’ energy requirements is proposed.

When the POI is monitored by more than one node, it causes unnecessary energy consumption by sending redundant data. In the future, we will consider how to adjust the energy requirement of the nodes to further balance their energy consumption. Specifically, on the basis of ECBTC, nodes can adjust the sensing range through local communication to avoid redundant data generation. On the other hand, these redundant data can also be reduced by performing suitable data fusion algorithms.

## Figures and Tables

**Table 1:** Symbols mentioned in Section 3.

Symbol	Definition	Unit
*N*	number of nodes	-
*T*	duration for the MC to perform a round of charging scheduling	s
*K*	number of time slices in a *T*	-
*p_k_*(*s_i_*)	energy consumption rate of *s_i_* in the *k*th time slice	Joule/s
*E^arr^*(*s_i_*)	residual energy of *s_i_* when the MC has just arrived	Joule
*E^o^*(*s_i_*)	energy that *s_i_* received from the MC	Joule
*O_j_*	set of nodes served by the MC during the *j*th traversal	-
*E_j_*(*MC*)	total energy consumption of the MC during the *j*th traversal	Joule
*T_j_*(*MC*)	total time consumption of the MC during the *j*th traversal	s
*p^m^*	energy consumption of the MC moving per unit of distance	Joule
*D_j_*	moving distance of the MC during the *j*th traversal	m
*p^c^*	energy transmission power of the MC	w
*T^c^*(*s_i_*)	duration for the MC to charge node *s_i_*	s
*v*	moving speed of the MC	m/s
*η*	charging efficiency of the MC	-
*λ_l_*	arrival intensity of event in POI*_l_*	-
*R*(*s_i_*)	sensing range of node *s_i_*	-
*ω*_1_	node energy consumption rate in absence of sensing event	w
*ω*_2_(*s_i_*)	node energy consumption rate of *s_i_* with the occurrence of sensing events	w

**Table 2:** Parameters mentioned in Section 4.

Symbol	Definition	Unit
*d*(*s_i_*, *s_p_*)	distance between *s_i_* and its parent *s_p_*	m
*d*_0_	the threshold distance	m
*P^t^*(*s_i_*, *s_p_*)	energy consumption rate of *s_i_* for sending	Joule/s
*P^r^*(*s_i_*)	energy consumption rate of *s_i_* for receiving	Joule/s
*e_elec_*	unit energy consumption of the circuit	nano-Joule
*ε_fs_*	constant parameters of the signal amplifier in the free space environment	-
*ε_amp_*	constant parameters of the signal amplifier in the multi-path fading environment	-
*d*(*s_i_*, *s_p_*)	distance between *s_i_* and its parent *s_p_*	m

**Table 3:** Types and number of items available for backpacks when *T* = 5 h and *K* = 5.

*ID*	*τ_y_*(*s_i_*)	*p_y_*(*s_i_*)	*m_y_*	Diagram
1	1	*ω*_1_	3	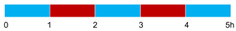
2	1	*ω*_2_(*s_i_*)	3	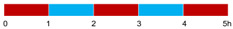
3	2	*ω*_1_	2	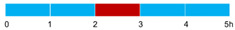
4	2	*ω*_2_(*s_i_*)	2	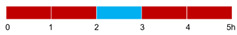
5	3	*ω*_1_	1	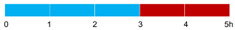
6	3	*ω*_2_(*s_i_*)	1	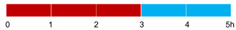
7	4	*ω*_1_	1	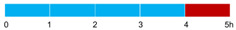
8	4	*ω*_2_(*s_i_*)	1	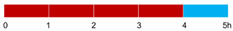
9	5	*ω*_1_	1	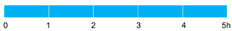
10	5	*ω*_2_(*s_i_*)	1	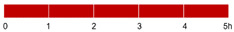

**Table 4:** Parameters mentioned in Section 5.

Symbol	Definition	Unit
*E_s_*	battery capacity of node	Joule
*T^c^*(*s_i_*)	duration for the MC to replenish *s_i_* in a *T*	s
*C^h^*	set of all nodes in the network that are *h* hops away from the BS	-
$\bar{d_{h}}$	mean Euclidean distance from the nodes in the set *C^h^* to the BS	m
*p^t^*(*d*)	power consumption of a node for transmitting data to a distance of *d* meters	w
*K*′	number of time intervals in a *T*	-
*DCN*(*s_i_*)	set of all children of *s_i_*	-
*P_k_*(*s_i_*)	probability that the energy consumption rate of *s_i_* is *p_k_*(*s_i_*) in the *k*th time slice	-

**Table 5:** Simulation parameters.

Symbol	Parameters	Values	Unit
*W* × *L*	network size	100 × 150	m^2^
*N*	number of nodes	50	-
*r_s_*	sensing radius	10	m
*r_c_*	communication radius	5	m
*B_packet_*	packet size	4	Kbit
*E_s_*	full energy of node	200	Joule
*e_elec_*	unit energy consumption of the circuit	50	nano-Joule
*ε_fs_*	constant parameters of the signal amplifier in the free space environment	0.01	-
*ε_amp_*	constant parameters of the signal amplifier in the multi-path fading environment	1.3 × 10^−6^	-
*E_MC_*	full energy of the MC	10^4^	Joule
*v*	moving speed of the MC	0.5	m/s
*p^m^*	energy consumption of the MC on moving per unit of distance	2	Joule
*p^c^*	energy transmission power of the MC	2	w
*η*	charging efficiency	0.8	-
*T*	duration for the MC to perform a round of charging scheduling	2820	s
*λ_l_*	arrival intensity of event in POI*_l_*	≤0.01	-

## Data Availability

Data is contained within the article.
